# The Institutional Worker

**Published:** 1912-05-18

**Authors:** 


THE
### INSTITUTIONAL WORKER
**Being a Special Supplement to "The Hospital"**

### Editor's Notices.

**Contributions:**
Contributions should be written, or preferably typed, on one side of the paper only, and all articles sent in are accepted upon the distinct understanding that they are forwarded to THE HOSPITAL only.

The Editor cannot undertake to return MSS. not used, but when a stamped directed envelope is enclosed the MSS. may be returned if a special request is made.

Accepted articles and paragraphs of news will be paid for after publication at the scale rate.

**Address:**
To prevent delay all contributions and letters on editorial business must be addressed exclusively to the Editor, "The Hospital," 29 Southampton Street, Strand, London, W.C. It is important that this regulation shall be strictly observed.

**Correspondence:**
Correspondence on all subjects is invited. The name and address of correspondents must be given as a guarantee of good faith, but not necessarily for publication.

**Special Articles:**
Special articles are invited, and questions, inquiries, and paragraphs upon all matters relating to the work, administration, and management of general, special, mental, fever, cottage and convalescent hospitals, sanatoria, homes, institutions, societies and organisations for the treatment or care of the sick, injured and dependants of all classes. Every matter affecting the interests and welfare of the staff of all grades working in these institutions will receive special consideration.

**Administrative Medicine, Research and Items of News:**
Special terms will be paid for approved articles on subjects in this wide field by experts who have made some section of it their special study and interest. We wish to give prominence to every movement tending to advance accuracy of diagnosis, efficiency in treatment, and the development of modern methods to eradicate disease and promote the welfare of the suffering.

**A Bureau of Information:**
We invite inquiries and applications for information and help in providing for that numerous class of sufferers who require special care or house-room which they cannot obtain in their own homes or secure for themselves. **We cannot, however, prescribe, or recommend practitioners.**

**Books for Review:**
Publishers are particularly requested to send advance proofs of any new books of importance whenever possible, as the Editor has made arrangements to publish immediate reviews on a new plan.

**Photographs, Plans, Blocks, and Illustrations:**
It is requested that wherever possible MSS. may be accompanied by illustrations in any of the above forms. The name of the sender and of the article to which it belongs should be written on the back of each photograph, drawing, or block for purposes of identification.

**Local Papers:**
Newspapers containing reports or news paragraphs **should be marked** and addressed to the Sub-Editor.

**The Coupon System:**
A coupon will be found at the bottom of the third inside page of the cover each week. This coupon must be attached to every question or inquiry to which an answer is desired in THE HOSPITAL.

### A Matter of Business.

ONLY a generation since the progress of a business was confined to the locality in which it was established. To-day, with the numerous facilities for effective publicity, a business may become world-wide, with a universal reputation. For the successful accomplishment of such an aim the press affords the most influential means, provided the media employed be judiciously selected.

If a business concerns any Profession or Trade, the most direct channels of approach to those interested are their own particular Journals, as these are associated exclusively with the peculiar requirements of the classes to whom they especially appeal.

Journals of this character are bought and read by all desiring assistance and authentic information in connection with their Profession or work, and consequently more personal value attaches to announcements that may appear within their pages.

To come under the direct notice, therefore, of those engaged in the Construction, Equipment, Administration, and Management of Institutions generally throughout this country, as well as the individual Worker in the Medical, Health, and Sanitary Services, it is necessary to employ THE HOSPITAL—the only Journal that covers the entire Institutional World.

The constant reiteration of a name or business notice week by week and month by month in a Journal of such recognised authority in its own direction, as THE HOSPITAL, must have a telling influence, create a deep and lasting impression, and result in considerable extension of business.

### Manager's Notices.

**All letters on business matters must be addressed to The Manager, "The Hospital," The Hospital Building, 28 and 29 Southampton Street, London, W.C.**

**Advertisements:**
To insure insertion, all advertisements for THE HOSPITAL must reach the Manager not later than Wednesday morning in each week. For scale of charges see pages v and 2.

**Special Rates** will be quoted for those Institutions that agree to send all their vacancy, contract, and public notices to THE HOSPITAL each year, so as to make it a file of such announcements for ready reference.

**Remittances:**
**It is especially requested that remittances be made by Cheque or Postal Order, and in halfpenny stamps only when the amount is under sixpence.**

**Subscriptions,** which may commence at any time, are payable in advance. Cheques and Post-Office Orders should be crossed London County and Westminster Bank, Covent Garden Branch, and made payable to the Manager of THE HOSPITAL.

RATES OF SUBSCRIPTION (payable in advance).

UNITED KINGDOM.

| | | |
|---|---|---|
| Three Months | (including Postage) ... ... ... ... ... | 2s. 0d. |
| Six Months | do. ... ... ... ... ... | 4s. 0d. |
| Twelve Months | do. ... ... ... ... ... | 6s. 6d. |

FOREIGN AND COLONIAL.

| | | |
|---|---|---|
| Three Months | (including Postage) ... ... ... ... ... | 2s. 6d. |
| Six Months | do. ... ... ... ... ... | 5s. 0d. |
| Twelve Months | do. ... ... ... ... ... | 8s. 8d. |

*SUBSCRIPTION FORM.*

*Please send me* THE HOSPITAL *for....................months, commencing with your issue of.............................., for which please find enclosed...........................*

*Name*...........................................................

*Address* ......................................................

.................................................................

*Date*..........................................

*To*...............................*Newsagent.*

### OFFICIAL APPOINTMENTS VACANT.

### Poor-Law Vacancies.

Assistant Matron (£20), Newport (Salop) Union. Mr. R. P. Liddle, C. to G., Newport, Salop. May 24.

Assistant Nurse (£25), Bishops Stortford Union. A. G. Gwynn, C. to G., Bishops Stortford. May 27.

Assistant Nurse (£20), Weymouth Union. Mr. H. A. Huxtable, C. to G., Weymouth. May 20.

Assistant Nurse (£25), Glanford Brigg Union. F. C. Hett, C. to G., 11 Bigby Street, Brigg. May 22.

Assistant Nurse (£30), Easington Union. J. M. Longden, C. to G., Union Offices, Easington, Castle Eden. May 23.

Assistant Nurses (£28), East Preston Union. Mr. A. Shelley, C. to G., Littlehampton. May 20.

Assistant Nurse (£20), Kingsclere Union. Mr. J. Barnes, C. to G., Newbury. May 27.

Charge Nurse (£30), Keighley Union. S. Green, C. to G., Keighley. May 27.

Charge Nurse (£30), Steyning Union. A. Flower, C. to G., Union Offices, Shoreham-by-Sea. May 27.

Charge Nurse (£30), Ecclesall Bierlow Union. J. E. Moulding, C. to G., Union Office, The Edge, Sheffield. May 29.

Charge Nurse (£35), Stoke-on-Trent Union. Mr. T. Wood, C. to G., Stoke-on-Trent Union. May 20.

Charge Nurse (£35), East Preston Union. Mr. A. Shelley, C. to G., Littlehampton. May 20.

Charge Nurse (£35), Bedwellty Union. Mr. H. J. C. Shepard, C. to G., Tredegar, Mon. May 21.

Charge Nurse (£30), Dudley Union. Mr. E. Allen, C. to G., Dudley. May 21.

Charge Nurse (32), Dorking Union. Mr. W. James, C. to G., Dorking. May 30.

Charge Nurse (£30), Epping Union. J. C. Archer, Acting C. to G., Epping. May 23.

Children's Attendant (£20), Lanchester Union. Mr. W. H. Ritson, C. to G., Lanchester. May 22.

Children's Nurse (£30), Merthyr Tydfil Union. F. T. James, C. to G., 134 High Street, Merthyr Tydfil. May 23.

Female Attendant (£26), Dudley Union. Mr. E. Allen, C. to G., Dudley. May 22.

